# Mutant prevention and minimum inhibitory concentration drug values for enrofloxacin, ceftiofur, florfenicol, tilmicosin and tulathromycin tested against swine pathogens *Actinobacillus pleuropneumoniae*, *Pasteurella multocida* and *Streptococcus suis*

**DOI:** 10.1371/journal.pone.0210154

**Published:** 2019-01-10

**Authors:** Joseph M. Blondeau, Shantelle D. Fitch

**Affiliations:** 1 Department of Clinical Microbiology, Royal University Hospital and the Saskatchewan Health Authority, Saskatoon, Saskatchewan, Canada; 2 Departments of Microbiology and Immunology, Pathology and Ophthalmology, University of Saskatchewan, Saskatoon, Saskatchewan, Canada; Natural Environment Research Council, UNITED KINGDOM

## Abstract

*Actinobacillus pleuropneumoniae*, *Pasteurella multocida* and *Streptococcus suis* are prevalent bacterial causes of swine infections. Morbidity, mortality and positively impacting the financial burden of infection occurs with appropriate antimicrobial therapy. Increasing antimicrobial resistance complicates drug therapy and resistance prevention is now a necessity to optimize therapy and prolong drug life. Mutant bacterial cells are said to arise spontaneously in bacterial densities of 10^7^−10^9^ or greater colony forming units/ml. Antibiotic drug concentration inhibiting growth of the least susceptible cell in these high density populations has been termed the mutant prevention concentration (MPC). In this study MPC and minimum inhibitory concentration (MIC) values of ceftiofur, enrofloxacin, florfenicol, tilmicosin and tulathromycin were determined against the swine pathogens *A*. *pleuropneumoniae*, *P*.*multocida* and *S*. *suis*. The following MIC_90_/MPC_90_ values (mg/L) for 67 *A*. *pleuropneumoniae* and 73 *P*. *multocida* strains respectively were as follows: *A*. *pleuropneumoniae* 0.031/0.5, ≤0.016/0.5, 0.5/2, 4/32, 2/32; *P*. *multocida* 0.004/0.25, 0.016/0.125, 0.5/0.5, 8/16, 0.5/1. For 33 *S*. *suis* strains, MIC_90_ values (mg/L) respectively were as follows: 1, 0.25, 4, ≥8 and ≥8. A total of 16 *S*. *suis* strains with MIC values of 0.063–0.5 mg/L to ceftiofur and 0.25–0.5 mg/L to enrofloxacin were tested by MPC; MPC values respectively were 0.5 and 1 mg/L respectively. MPC concentrations provide a dosing target which may serve to reduce amplification of bacterial subpopulations with reduced antimicrobial susceptibility. Drug potency based on MIC_90_ values was ceftiofur > enrofloxacin >florfenicol = tulathromycin > tilmicosin; based on MPC_90_ values was enrofloxacin > ceftiofur > tulathromycin > florfenicol ≥ tilmicosin.

## Introduction

Bacterial infectious diseases are complicated by antimicrobial resistance and global concerns with the clinical impact of resistance is redefining antimicrobial utilization [1,2]. In addition to clinical outcomes, microbiological measurements continue to contribute to novel data on drug use for treatment and bacterial eradication. Optimization of therapy needs to consider clinical outcomes and antimicrobial resistance prevention during therapy. Guideline documents for antimicrobial therapy of human infectious diseases give consideration of antimicrobial agents with a reduced likelihood for resistance selection to be important when making therapeutic choices [3,4]. Such an observation clearly impacts economic costs and adds substantially to treatment costs.

In pigs, respiratory disease is amongst the most important health concerns for swine producers. Swine respiratory disease has been previously recognized as the main pathogen-identified cause of swine mortality accounting for deaths in ~44% of nursing pigs and ~61% of grown finished pigs [5]. Porcine respiratory disease complex (PRDC) is a multifactorial clinical entity describing pneumonia in pigs where multiple etiologies contribute to the pathogenesis leading to clinical disease [5]. This complex etiology and pathogenesis may include one or more viruses, *Mycoplasma hyopneumoniae*, opportunistic and pathogenic bacteria. *P*. *multocida* is an important pig pathogen and is carried by a large number of animals and transmission is mostly by aerosols [6,7]. Following invasion, *P*. *multocida* multiplies quickly, liberates toxins and causes necrotic lesions in lung tissue. *A*. *pleuropneumoniae* is highly contagious and causes an acute or chronic fibro-haemorrhagic necrotising pneumonia [8]. Ceftiofur (beta-lactam), enrofloxacin (fluoroquinolone), florfenicol (phenol), tilmicosin (macrolide) and tulathromycin (triamalide) are commonly used for swine infections.

Antimicrobial susceptibility or resistance is determined *in vitro* by measuring the minimum inhibitory concentration (MIC) utilizes a bacterial inoculum of 10^5^ colony forming units per millilitre (cfu/ml) [9]. Previous publications for human infectious diseases reported substantially higher bacterial densities (i.e. 10^7^−10^9^ cfu/ml or higher) during infections such as meningitis, pneumonia and from protected brush specimens from patients with an acute bacterial exacerbation of their chronic lung disease [10–13]. Additionally, McVey and Kusak studied lung, tonsil and trachea tissues from calves with bronchopneumonia and reported 12% of samples had >10^8^ cfu/g and 50% of specimens hads >10^5^ cfu/g with *Mannheimia haemolytica* being the most common organism recovered [14]. Given the substantially higher bacterial densities in infection than tested in an MIC assay, it begs the question as to the true dynamics of bug/drug interactions when higher bacterial densities are encountered. The mutant prevention concentration (MPC) describes a drug concentration threshold or lowest drug concentration blocking growth of mutant bacterial sub-populations [15,16] that spontaneously arise in bacterial densities of 10^7^−10^9^ cfu–densities seen with infection. Antibiotic drug concentrations insufficiently inhibiting mutant cell growth result in selective amplification of bacterial cells with reduced drug susceptibility [16,17]. In a study with fluoroquinolones and the human pathogen *Streptococcus pneumoniae*, differences were seen between fluoroquinolone compounds and macrolide compounds and MPC values [18,19]. Published MPC studies have been completed with human pathogens and fluoroquinolones, macrolides and many other drug classes [17,18,20–22]. Fluoroquinolones used in veterinary medicine were previously tested by MPC against *E*. *coli* and *Staphylococcus pseudintermedius* and MPC measurements with *M*. *haemolytica* have been reported for ceftiofur, enrofloxacin, florfenicol, tilmicosin and tulathromycin showing differences between compounds in their ability to prevent mutant growth at clinically relevant concentrations [17,23,24].

Here we report on testing of swine clinical isolates of *A*. *pleuropneumoniae*, *P*. *multocida* and *S*. *suis* by MPC to ceftiofur, enrofloxacin, florfenicol, tilmicosin and tulathromycin to determine antimicrobial drug concentrations blocking the most resistant bacterial organisms in high density cultures. Observations reported here may inform thinking on antimicrobial use to affect clinical cure, minimize resistance selection during therapy and pharmacokinetic/pharmacodynamic modelling.

## Materials and methods

### Bacterial strains

Bacterial pathogens collected from swine in the U.S.A were used: *A*. *pleuropneumoniae* (n = 67), *P*. *multocida* (n = 73), *S*. *suis* (n = 59). These organisms were generously provided by Dr. Ching Ching Wu from the Indiana Animal Disease Diagnostic Laboratory, Purdue University, West Lafayette, Indiana. Bacterial strains were identified by Vitek II (bioMerieux, St. Laurent, QC), matrix assisted laser desorption ionization-time of flight (MALDI-TOF) (bioMerieux, St. Laurent, QC) and/or biochemical tests as summarized in the Manual of Clinical Microbiology [25]. Individual strains were stored at -70°C in skim milk. For MIC testing, bacteria were thawed and subcultured two times on blood agar (tryptic soy agar containing 5% sheep red blood cells) (BA) plates with incubation for 18–24 hours at 35–37°C in oxygen (O_2_). Bacterial strains included in the study needed to be susceptible (where breakpoints exist) to the drugs tested by interpretative criteria as per the Clinical and Laboratory Standards Institute (CLSI) [9].

### Antimicrobial compounds

Enrofloxacin was provided by Bayer Animal Health, Shawnee Mission, Kansas esd prepared as per manufacturer’s instructions. Ceftiofur, florfenicol, tilmicosin and tulathromycin were purchased commercially through the Western College of Veterinary Medicine Pharmacy at the University of Saskatchewan and reconstituted based on manufacturer’s directions. Fresh stock solutions or those prepared from frozen samples (-70°C) were used. For quality control, the following American Type Culture Collection (ATCC) control strains were included in each susceptible assay to ensure performance of the susceptibility assays: *Enterococcus faecalis* ATCC 29212, *Pseudomonas aeruginosa* ATCC 27853, *E*. *coli* ATCC 25222, *Staphylococcus aureus* ATCC 29213. MIC values needed to be within acceptable ranges for each organism/drug.

### MIC measurements

MIC testing was based on the recommended CLSI procedure [9]. Briefly, Mueller-Hinton broth (MHB) containing two-fold concentration of drug was added to 96-well micro-dilution trays. A 0.5 McFarland density of *A*. *pleuropneumoniae*, *P*. *multocida* and *S*. *suis* was further diluted to 5 x 10^5^ cfu/ml, added to the microdilution tray containing drug and incubated for 18–24 hours (35–37°C) in O_2_. The MIC was interpreted as the lowest drug concentration inhibiting visible growth. The designation of MIC_50_ and MIC_90_ are determined by calculating the drug concentration inhibiting 50% or 90% of strains respectfully by starting from the lowest MIC or MPC values.

### MPC testing

MPC testing was adapted from the method published for *S*. *pneumoniae* and previously reported for *Mannheimia haemolytica* [18,24]. Starter cultures for *A*. *pleuropneumoniae* and *P*. *multocida* were inoculated on 5 BA plates per isolate to produce confluent growth and then incubated at 35–37°C for 18–24 hours in O_2_. Starter cultures for *S*. *suis* isolates were on 5 chocolate agar plates with incubation for 18–24 hours at 35-37°C in O_2_ following which the plate surfaces were swabbed to remove bacterial growth and transferred to 100 ml of brain heart infusion broth containing nicotinamide adenine dinucleotide (NAD) (*A*. *pleuropneumoniae*) or MHB (*P*. *multocida*) or Veterinary Fastidious Medium (MHB plus 3% laked horse blood) (Trek Diagnostic System, Cleveland, Ohio) (*S*. *suis*) and incubated as described. Following incubation, turbidity measurements verified cell densities of 3 x 10^8^ cfu/ml. Centrifugation at 5000 x G for 3 minutes at 4°C was used to concentrate bacteria following which the pellet was added to 3 ml of fresh medium. Drug containing agar plates (7 drug concentrations in doubling dilution) were inoculated with 200 ul (10^10^ cfu) of bacterial suspension and incubated for 24 hours at 35-37°C in O_2_ and screened for growth. Plates were reincubated for an additional 24 hours and the final reading recorded. The MPC value was the lowest drug concentration blocking growth. Drug concentrations tested were ceftiofur 0.06 to 4 mg/L, enrofloxacin 0.004 to 2 mg/L, florfenicol 0.5 to 32 mg/L, tilmicosin 0.5 to 64 mg/L, tulathromycin 0.25 to 16 mg/L. The designation of MPC_50_ and MPC_90_ are by calculating the drug concentration inhibiting 50% or 90% of strains respectfully by starting from the lowest MIC or MPC values.

## Results

MIC and MPC data for *A*. *pleuropneumoniae* strains and the 5 drugs is shown in Table 1. Drug concentrations inhibiting 50% and 90% respectively of bacterial strains is the MIC_50_ and MIC_90_ or MPC_50_ and MPC_90_ depending on the *in vitro* measurement. For ceftiofur, MIC_range,_ MIC_50_ and MIC_90_ and values were ≤0.016–0.063 mg/L, 0.016 mg/L and 0.031 mg/L; for enrofloxacin ≤0.016 mg/L, <0.016 mg/L, and ≤0.016; for florfenicol 0.5–1 mg/L, 0.25 mg/L and 0.5 mg/L; for tilmicosin 1–8 mg/L, 2 mg/L and 4 mg/L; for tulathromycin 0.5–8 mg/L, 1 mg/L and 2 mg/L. A comparison of MPC values are also shown in Table 1 for the *A*. *pleuropneumoniae* strains. The MPC_range_, MPC_50_ and MPC_90_ values were as follows respectively for each agent: ceftiofur ≤0.016–1 mg/L, 0.063 mg/L, 0.5 mg/L; enrofloxacin 0.063–0.5 mg/L, 0.125 mg/L, 0.5 mg/L; florfenicol 0.25–4 mg/L, 0.5 mg/L, 2 mg/L; tilmicosin 8–64 mg/L, 32 mg/L, 32 mg/L; tulathromycin 8–32 mg/L, 32 mg/L, 32 mg/L.

**Table 1:** MIC and MPC values for 67 *A*. *pleuropneumoniae* isolates from swine.

Drug	MIC/MPC Distribution Values (mg/L)													
	≤0.016	0.031	0.063	0.125	0.25	0.5	1	2	4	8	16	32	≥64	
**MIC Distribution**														**MIC**_**50/90**_
Ceftiofur	46	19	2											≤0.016/0.031
Enrofloxacin	67													≤0.016/≤0.016
Florfenicol					30	36	1							0.5/0.5
Tilmicosin							2	49	15	1				2/4
Tulathromycin						5	54	7		1				1/2
**MPC Distribution**														**MPC**_**50/90**_
Ceftiofur	2	15	26	11	5	2	6							0.063/0.5
Enrofloxacin			25	18	14	10								0.125/0.5
Florfenicol					1	48	9	6	3					0.5/2
Tilmicosin										11	13	41	2	32/32
Tulathromycin										1	10	56		32/32

Table 2 summarizes MIC and MPC data for the *P*. *multocida* strain tested against the 5 drugs investigated. The MIC_range_, MIC_50_ and MIC_90_ values respectively for each agent were as follows: ceftiofur ≤0.016–0.031 mg/L, ≤0.016 mg/L, ≤0.016 mg/L; enrofloxacin ≤0.016 mg/L, ≤0.016 mg/L, ≤0.016 mg/L; florfenicol 0.25–1 mg/L, 0.5 mg/L, 0.5 mg/L; tilmicosin 1–8 mg/L, 2 mg/L, 4 mg/L; tulathromycin 0.063–1 mg/L, 0.25 μ/ml, 0.5 mg/L. MPC_range_, MPC_50_ and MPC_90_ values respectively were as follows: ceftiofur 0.031–0.5 mg/L, 0.125 mg/L, 0.25 mg/L; enrofloxacin ≤0.016–0.125 mg/L, 0.063 mg/L, 0.125 mg/L; florfenicol 0.25–2 mg/L, 1 mg/L, 1 mg/L; tilmicosin 2-≥64 mg/L, 8 mg/L, 16 mg/L; tulathromycin 0.5–8 mg/L, 1 mg/L, 1 mg/L.

**Table 2:** MIC and MPC values for 73 *P*. *multocida* isolates from swine.

Drug	MIC/MPC Distribution Values (mg/L)													
	≤0.016	0.031	0.063	0.125	0.25	0.5	1	2	4	8	16	32	≥64	
**MIC Distribution**														**MIC**_**50/90**_
Ceftiofur	72	1												≤0.016/≤0.016
Enrofloxacin	73													≤0.016/≤0.016
Florfenicol					21	48	4							0.5/0.5
Tilmicosin							7	35	25	4				2/4
Tulathromycin			1	9	38	21	4							0.25/0.5
**MPC Distribution**														**MPC**_**50/90**_
Ceftiofur		14	16	22	17	1								0.125/0.25
Enrofloxacin		19	34	15										0.063/0.125
Florfenicol					1	12	59	1						1/1
Tilmicosin								2	13	38	15	4	1	8/16
Tulathromycin						19	47	3	3	1				1/1

A total of 59 *S*. *suis* strains (Table 3) had MICs of 0.031 to 2 mg/L for ceftiofur with an MIC_50_ and MIC_90_ of 0.063 mg/L and 1 mg/L; for enrofloxacin values ranged from 0.063-≥4 mg/L with an MIC_50_ of 0.05 mg/L and an MIC_90_ of 0.5 mg/L. For florfenicol, MICs ranged from 2-≥4 mg/L and a MIC_50_ and MIC_90_ of ≥4 mg/L. All 59 strains had MIC values to tilmicosin and tulathromycin of ≥4 mg/L with MIC_50_ and MIC_90_ values of 8 mg/L. For MPC testing, 16 *S*. *suis* strains with MIC to ceftiofur of 0.063–0.5 mg/L were tested and MPC values were 0.124 (n = 7), 0.25 (n = 5) and 0.5 (n = 4) mg/L with MPC_50_ and MPC_90_ values of 0.25 mg/L and 0.5 mg/L. By comparison, 12 *S*. *suis* strains with MIC to enrofloxacin of 0.125–0.25 mg/L had MPC values of 0.25 (n = 1), 0.5 (n = 1), 1 (n = 8), 2 (n = 1) and 4 mg/L (n = 1) with an MPC_50_ and MPC_90_ of 1 mg/L. MPC testing against florfenicol, tilmicosin and tulathromycin was not done due to the high (≥4mg/L) MIC values.

**Table 3:** Comparative MIC values for 59 *S*. *suis* strains collected from swine.

Drug	MIC Distribution Values (mg/L)									MIC_50/90_
	0.031	0.063	0.125	0.25	0.5	1	2	4	≥8	
Ceftiofur	6	29	3	1	3	3	4	1	9	0.063/1
Enrofloxacin		1	4	28	23	1		2		0.25/0.5
Florfenicol							29	30*		≥4/≥4
Tilmicosin						1			58	≥4/≥4
Tulathromycin									59	≥4/≥4

*≥4 mg/L

By MPC testing, no strains of *A*. *pleuropneumoniae* or *P*. *multocida* had values ≥2 mg/L for ceftiofur. For enrofloxacin, no strains of *P*. *multocida* had MPC values ≥0.25 mg/L, however, 10 strains of *A*. *pleuropneumoniae* had MPCs of 0.5 mg/L (breakpoint ≤0.25 mg/L). Three strains of *A*. *pleuropneumoniae* had MPC values of 4 mg/L to florfenicol (≤2 mg/L breakpoint). For tilmicosin (≤16 mg/L breakpoint) 43/67 (64.1%) *A*. *pleuropneumoniae* strains had a MPC value of ≥32 mg/L compared to 5/73 (6.8%) *P*. *multocida* strains with MPC values ≥32 mg/L. For tulathromycin with a ≤64 mg/L breakpoint for *A*. *pleuropneumoniae* and ≤16 mg/L breakpoint for *P*. *multocida*, no *A*. *pleuropneumoniae* strains had MPC values of ≥64 mg/L and no strains of *P*. *multocida* had MPC values ≥16 mg/L.

## Discussion

This is the first report of MPC results for *A*. *pleuropneumoniae* and *P*. *multocida* swine pathogen clinical isolates tested against veterinary approved drugs including fluoroquinolones, beta-lactams, phenols, macrolides and triamalide drug classes; MPC values were lowest for ceftiofur and enrofloxacin. Lei *et al* previously reported an MPC value for florfenicol against *S*. *suis* strains of 3.2 μg/ml and the MIC_90_ was 2 μg/ml [26]. Florfenicol MPC values against the *S*. *suis* strains in our study were not determined nor were those for tilmicosin or tulathromycin due to the high MIC values. As with previous publications with human and veterinary pathogens and antimicrobials, MPC values were higher than MIC values [17,27]. This study adds further to the growing body of MPC data and further confirms MPC measurements for important veterinary pathogens and antimicrobial agents. Dorey *et al* recently commented on the lack of published data for swine pathogens detailing MIC, MPC and MSW and how such data was necessary for PK/PD modelling studies [28].

The *in vitro* activity of antimicrobials used in swine on *A*. *pleuropneumoniae*, *P*. *multocida* and *s*.*suis* has been previously reported. Salmon *et al* and Portis *et al* reported MIC_50_ and MIC_90_ values for ceftiofur and enrofloxacin that were consistent overall with values in this report, particularly for the *A*. *pleuropneumoniae* and *P*. *multocida* strains [29,30]. Florfenicol, tilmicosin and tulathromycin MIC_90_ values were higher in the publication of Portis *et al* than in our report and this most likely is due to that study being more a surveillance report whereas in our study we selected strains with MICs at or below susceptibility breakpoints where available. Shin *et al* reported on MIC_90_ values of 0.5 μg/ml for florfenicol tested against *A*. *pleuropneumoniae* and *P*. *multocida* strains [31].

MPC investigations with various classes of antimicrobial agents have been reported [17,22,24,32] despite an earlier publication suggesting MPC measurements only apply to fluoroquinolones and not aminoglycosides, macrolides or beta-lactams [33]. For example, Metzler *et al* compared MPC values for azithromycin, clarithromycin and erythromycin against *S*. *pneumoniae* strains and showed clarithromycin was statistically less likely to select for organism with reduced susceptibility and azithromycin was statistically more likely [19].

A number of publications have investigated pharmacological modelling of the mutant selection window and dosing strategies that fall within or outside of the MSW [34–37]. The mutant selection window (MSW) is bordered by the MIC (lower drug concentration) and the MPC (upper drug concentration). Firsov *et al* tested the MSW hypothesis using *S*. *aureus*, daptomycin and vancomycin and found that selection of organisms resistant to 2X and 4X the MIC of either drug occurred with antibiotic concentrations falling within the MSW [36], a finding supporting the MSW hypothesis. Similar findings were reported with fluoroquinolones and *S*. *aureus* strains [34]. From investigators with *Streptococcus pneumoniae* and moxifloxacin, resistance was selected at drug concentrations falling within the MSW and a more recent report found that time within the MSW was an appropriate prediction of bacterial resistance [35,38]. In a report from testing gatifloxacin and *S*. *pneumoniae* in a rabbit empyema model, dosing of gatifloxacin to remain within the MSW for ≥40% of the dose resulted in mutant subpopulation amplification [39].

We [24] and others [40,41]have previously commented on some of the characteristics of macrolide and macrolide like compounds (azalides, triamilides) in human and veterinary medicine. In particular, all such agents have low serum drug concentrations and lung concentrations vary when considering epithelial lining fluid drug concentrations versus alveolar macrophage drug concentrations. Our susceptibility results for tulathromycin appear consistent with results from previous measurements with *M*. *haemolytica* and more recently *P*. *multocida* [24,42]. In this study, the MIC_90_ values for tilmicosin were 4 mg/L against the *A*. *pleuropneumoniae* and *P*. *multocida* strains as compared to 2 and 0.5 mg/L respectively for tulathromycin. MIC values in excess of 0.5 mg/L are above achievable or sustainable blood concentrations for tilmicosin and tulathromycin (www.zoetis.com).

For both tilmicosin and tulathromycin, MPC values were ≥8 mg/L for all strains of *A*. *pleuropneumoniae*. For tilmicosin MPC values were ≥2 mg/L for all *P*. *multocida* strains as compared to ≥0.5 mg/L for tulathromycin. For those compounds and considering the MSW and MIC_90_ and MPC_90_ values, the MSW for tilmicosin and *A*. *pleuropneumoniae* would range from 4 to 32 mg/L (8-fold difference) and for *P*. *multocida* from 4 to 16 mg/L (4-fold difference). By comparison, for tulathromycin and *A*. *pleuropneumoniae* the MSW would range from 2–32 mg/L (16-fold difference) and for *P*. *multocida* the MSW was narrow being the same or within a 2-fold difference. For individual strains, the MSW may be narrower if the fold difference between the measured MIC and MPC values were less.

In swine lungs, tulathromycin concentrations peak at approximately 3.5 mg/L [43,44]. Tulathromycin neutrophil and alveolar macrophage concentrations in pigs were 16.6 and 8.1 x respectively the extracellular fluid concentration [44,45]. Based on MIC and MPC values reported in this study, pulmonary drug concentrations for tulathromycin would fall within the MSW for the *A*. *pleuropneumoniae* and *P*. *multocida* strains. For the *A*. *pleuropneumoniae* strains, all 67 strains had MPC values (8–32 mg/L) above the maximum pulmonary drug concentration of 3.5 mg/L and for *P*. *multocida* strains 4/73 (5.4%) had MPC values above the maximum pulmonary drug concentration. Based on the data of Benchaoui *et al*, time within the MSW for tulathromycin for *A*. *pleuropneumoniae* and *P*. *multocida* could be as long as 15 days due to the long elimination half-life.[43] Given the achievable serum and pulmonary concentrations for tulathromycin, the susceptible breakpoints of 16 mg/L and 64 mg/L for *P*. *multocida* and *A*. *pleuropneumoniae* respectively is puzzling.

For the remaining drugs tested and considering the MSW, blood levels of ceftiofur exceed the MSW for 48–6 hours but we did not conduct testing to account for the high protein binding (>90%) associated with this compound. Others have shown the protein binding in excess of 60% elevates MIC values *in vitro* when protein is included in the susceptibility assays [46–48]. How this observation translates clinically is not fully understood. Enrofloxacin (7.5 mg/kg) blood levels exceed the MSW for ~12–18 hours for isolates with MPCs of 0.5 mg/L. In our study, 71% of isolates had MPC values to enrofloxacin ≤0.5 mg/L. Considering florfenicol, blood concentrations fall within the MSW, however, MPC values of ≤4 mg/L were seen for the majority of isolates tested and for these, drug concentration would exceed the MSW for ~6 hours.

The MPC defines an *in vitro* measurement using high density bacterial populations that are representative of bacterial burdens present in acute infections. The MSW provide a drug concentration range where therapeutic drug concentrations falling and remaining in this range, based on approved dosage may allow for selective amplification of the least susceptible cells in the population. Dosing to achieve or exceed the MPC and hence the MSW prevents growth of bacterial cells with reduced susceptibility, however, dosing to exceed the MSW does not appear possible for all bug-drug combinations. In our study, enrofloxacin and ceftiofur had lower MIC and MPC values than did the other drugs. MPC principles may optimize therapy and impact resistance while providing valuable data for pharmacokinetic and pharmacodynamic modelling. Optimization of therapy while minimizing the potential for antimicrobial resistance are major principals for antimicrobial stewardship [49].

## References

[1] Nicolau DP. Optimizing outcomes with antimicrobial therapy through pharmacodynamic profiling. Journal of Infection and Chemotherapy. 2003; 9: 292–296. 10.1007/s10156-003-0279-x 14691648

[2] Dagan R, Klugman KP, Craig WA, Baquero F. Evidence to support the rationale that bacterial eradication in respiratory tract infection is an important aim of antimicrobial therapy. Journal of Antimicrobial Chemotherapy. 2001; 47: 129–140. 11157895 10.1093/jac/47.2.129

[3] Mandell LA, Wunderink RG, Anzueto A, Bartlett JG, Campbell GD, Dean NC, et al Infectious Disease Society of America/American Thoracic Society Consensus Guidelines on the management of community-acquired pneumonia in adults. Clinical Infectious Diseases. 2007; 44: S27–S72. 10.1086/511159 17278083 PMC7107997

[4] Solomkin JS, Mazuski JE, Bradley JS, Rodvold KA, Goldstein EJC, Baron EJ, et al Diagnosis and management of complicated intra-abdominal infection in adults and children: guidelines by the Surgical Infection Society and the Infectious Diseases Society of America. Clinical Infectious Diseases. 2010; 50: 133–164. 10.1086/649554 20034345

[5] Dosen R, Prodanov J, Milanov D, Stojanov I, Pusic I. The bacterial infections of respiratory tract of swine. Biotechnology in Animal Husbandry. 2007; 23: 237–243.

[6] Kamp EM, Bokken GC, Vermeulen TM, de Jong MF, Buys HE, Reek FH, et al A specific and sensitive PCR assay suitable for large-scale detection of toxigenic Pasteurella multocida in nasal and tonsillar swabs specimens of pigs. Journal of Veterinary Diagnostic Investigation. 1996; 8: 304–309. 10.1177/104063879600800305 8844572

[7] Bochev I. Porcine respiratory disease complex (PRDC): A review. I Etiology, epidemiology, clinical forms and pathoanatomical features. Bulgarian Journal of Veterinary Medicine. 2007; 10: 131–146.

[8] Sebunya TN, Saunders JR. Haemophilus pleuropneumoniae infection in swine: a review. Journal of the American Veterinary Medical Association. 1983; 182: 1331–1337. 6348003

[9] Clinical and Laboratory Standards Institute (2013) Performance standards for antimicrobial disk and dilution susceptibility tests for bacteria isolated from animals: approved standard—fourth edition (VET01-A4).

[10] Bingen E, Lambert-Zechovsky N, Mariani-Kurkdjian P, Doit C, Aujard Y, Fournerie F, et al Bacterial counts in cerebrospinal fluid of children with meningitis. European Journal of Clinical Microbiology and Infectious Diseases. 1990; 9: 278–281. 2112465 10.1007/BF01968060

[11] Feldman W. Concentrations of bacteria in cerebrospinal fluid of patients with bacterial meningitis. Journal of Pediatrics. 1976; 88: 549–552. 3635 10.1016/s0022-3476(76)80003-0

[12] Frisch AW, Tripp JT, Barrett CD Jr., Pidgeon BE. The specific polysaccharide content of pneumonic lungs. Journal of Experimental Medicine. 1942; 76: 505–510. 19871253 10.1084/jem.76.6.505 PMC2135280

[13] Fagon J, Chastre J, Trouillet JL, Domart Y, Dombret MC, Bornet M, et al Characterization of distal bronchial microflora during acute exacerbation of chronic bronchitis. Use of the protected specimen brush technique in 54 mechanically ventilated patients. American Review of Respiratory Disease. 1990; 142: 1004–1008. 10.1164/ajrccm/142.5.1004 2240819

[14] McVey DS, Kuszak J. Bacterial isolates from the lungs of beef calves with bronchopneumonia associated with acute bovine respiratory disease.; 2010 December 4–5; Chicago, IL.

[15] Dong Y, Zhao X, Domagala J, Drlica K. Effect of fluoroquinolone concentration on selection of resistant mutants of *Mycobacterium bovis* BCG and *Staphylococcus aureus*. Antimicrobial Agents and Chemotherapy. 1999; 43: 1756–1758. 10390236 10.1128/aac.43.7.1756 PMC89357

[16] Blondeau JM, Hansen GT, Metzler KL, Hedlin P. The role of PK/PD parameters to avoid selection and increase of resistance: mutant prevention concentration. Journal of Chemotherapy. 2004; 16: 1–19. 15334827 10.1080/1120009x.2004.11782371

[17] Blondeau JM. New concepts in antimicrobial susceptibility testing: the mutant prevention concentration and mutant selection window approach. Veterinary Dermatology. 2009; 20: 383–396. 10.1111/j.1365-3164.2009.00856.x 20178475

[18] Blondeau JM, Zhao X, Hansen GT, Drlica K. Mutant prevention concentrations (MPC) of fluoroquinolones for clinical isolates of Streptococcus pneumo*niae*. Antimicrobial Agents and Chemotherapy. 2001; 45: 433–438. 10.1128/AAC.45.2.433-438.2001 11158737 PMC90309

[19] Metzler KL, Drlica K, Blondeau JM. Minimal inhibitory and mutant prevention concentrations for azithromycin, clarithromycin and erythromycin with clinical isolates of *Streptococcus pneumoniae*. Journal of Antimicrobial Chemotherapy. 2013; 68: 631–635. 10.1093/jac/dks461 23169894 PMC3566671

[20] Hansen GT, Blondeau JM. Comparison of the minimum inhibitory, mutant prevention and minimum bactericidal concentrations of ciprofloxacin, levofloxacin and garenoxacin against enteric gram-negative urinary tract infection pathogens. Journal of Chemotherapy. 2005; 17: 484–492. 10.1179/joc.2005.17.5.484 16323436

[21] Hedlin P, Blondeau JM. Comparative minimal inhibitory and mutant prevention drug concentrations of four fluoroquinolones against ocular isolates of Haemophilus influenzae. Eye & Contact Lens. 2007; 33: 161–164. 17502752 10.1097/01.icl.0000246872.73559.43

[22] Hesje CK, Drlica K, Blondeau JM. Mutant prevention concentration of tigecycline for clinical isolates of *Streptococcus pneumoniae* and *Staphylococcus aureus*. Journal of Antimicrobial Chemotherapy. 2015; 70: 494–497. 10.1093/jac/dku389 25324419 PMC4291236

[23] Wetzstein HG. Comparative mutant prevention concentrations of pradofloxacin and other veterinary fluoroquinolones indicate differing potentials in preventing selection of resistance. Antimicrobial Agents and Chemotherapy. 2005; 49: 4166–4173. 10.1128/AAC.49.10.4166-4173.2005 16189094 PMC1251556

[24] Blondeau JM, Borsos S, Blondeau LD, Blondeau BJ, Hesje C. Comparative minimum inhibitory and mutant prevention drug concentrations of enrofloxacin, ceftiofur, florfenicol, tilmicosin and tulathromycin against bovine isolates of *Mannheimia haemolytica*. Veterinary Microbiology. 2012; 160: 85–90. 10.1016/j.vetmic.2012.05.006 22677482

[25] Zbinden R (2015) Aggregatibacter, Capnocytophaga, Eikenella, Kingella, Pasteurella and other fastidious or rarely encountered Gram-negative rods Manual of Clinical Microbiology. 11th ed Washington, DC: American Society of Microbiology pp. 652–666.

[26] Lei Z, Liu Q, Yang S, Yang B, Khaliq H, Li K, et al PK-PD integration modeling and cutoff value of florfenicol against *Streptococcus suis* in pigs. Frontiers in Pharmacology. 2018; 9: 1–12. 10.3389/fphar.2018.00001 29387013 PMC5776115

[27] Hesje C, Borsos S, Blondeau JM. Determination of the minimum inhibitory concentration and mutant prevention concentration (MPC) of tigecycline against clinical isolates of Streptococcus pneumoniae: impact of media on MPC results.; 2007 March 31-April 3; Munich, Germany.

[28] Dorey L, Pelligand L, Cheng Z, Lees P. Pharmacokinetic/pharmacodynamic integration and modelling of florfenicol for the pig pneumonia pathogens Actinobacillus pleuropneumoniae and Pasteurella multocida. PLoS One. 2017; 12: e0177568 10.1371/journal.pone.0177568 28552968 PMC5446118

[29] Salmon SA, Watts JL, Case CA, Hoffman LJ, Wegener HC, Yancey RJ Jr. Comparison of MICs of ceftiofur and other antimicrobial agents against bacterial pathogens of swine from the United States, Canada, and Denmark. J Clin Microbiol. 1995; 33: 2435–2444. 7494042 10.1128/jcm.33.9.2435-2444.1995 PMC228432

[30] Portis E, Lindeman C, Johansen L. Antimicrobial susceptibility of porcine Pasteurella multocida, Streptococcus suis, and Actinobacillus pleuropneumoniae from the United States and Canada, 2001 to 2010. Journal of Swine Health and Production. 2013; 21: 30–41.

[31] Shin SJ, Kang SG, Nabin R, Kang ML, Yoo HS. Evaluation of the antimicrobial activity of florfenicol against bacteria isolated from bovine and porcine respiratory disease. Vet Microbiol. 2005; 106: 73–77. 10.1016/j.vetmic.2004.11.015 15737475

[32] Cui JC, Liu YN, Chen LA. Mutant prevention concentration of tigecycline for carbapenem-susceptible and -resistant *Acinetobacter baumannii*. Journal of Antibiotics. 2010; 63: 29–31. 10.1038/ja.2009.111 19911030

[33] Smith H, Nichol KA, Hoban DJ, Zhanel GG. Stretching the mutant prevention concentration (MPC) beyond its limits. Journal of Antimicrobial Chemotherapy. 2003; 51: 1323–1325. 10.1093/jac/dkg255 12716780

[34] Firsov AA, Vostrov SN, Lubenko IY, Drlica K, Portnoy YA, Zinner SH. *In vitro* pharmacodynamic evaluation of the mutant selection window hypothesis: four fluoroquinolones against *Staphylococcus aureus*. Antimicrobial Agents and Chemotherapy. 2003; 47: 1604–1613. 10.1128/AAC.47.5.1604-1613.2003 12709329 PMC153314

[35] Firsov AA, Portnoy YA, Strukova EN, Shlykova DS, Zinner SH. Predicting bacterial resistance using the time inside the mutant selection window: possibilities and limitations. International Journal of Antimicrobial Agents. 2014; 44: 301–305. 10.1016/j.ijantimicag.2014.06.013 25218155

[36] Firsov AA, Smirnova MV, Lubenko IY, Vostrov SN, Portnoy YA, Zinner SH. Testing the mutant selection window hypothesis with *Staphylococcus aureus* exposed to daptomycin and vancomycin in an in vitro dynamic model. Journal of Antimicrobial Chemotherapy. 2006; 58: 1185–1192. 10.1093/jac/dkl387 17028094

[37] Drlica K, Zhao X. Mutant selection window hypothesis updated. Clinical Infectious Diseases. 2007; 44: 681–688. 10.1086/511642 17278059

[38] Zinner SH, Lubenko IY, Gilbert DN, Simmons K, Zhao X, Drlica K. Emergence of resistant *Streptococcus pneumoniae* in an *in vitro* dynamic model that simulates moxifloxacin concentrations inside and outside the mutant selection window: related changes in susceptibility, resistance frequency and bacterial killing. Journal of Antimicrobial Chemotherapy. 2003; 52: 616–622. 10.1093/jac/dkg401 12951352

[39] Croisier D, Etienne M, Bergoin E, Charles P-E, Lequeu C, Piroth L, et al Mutant selection window in levofloxacin and moxifloxacin treatments of experimental pneumococcal pneumonia in a rabbit model of human therapy. Antimicrobial Agents and Chemotherapy. 2004; 48: 1699–1707. 10.1128/AAC.48.5.1699-1707.2004 15105123 PMC400524

[40] Nowakowski MA, Inskeep PB, Risk JE, Skogerboe TL, Benchaoui HA, Meinert TR, et al Pharmacokinetics and lung tissue concentrations of tulathromycin, anew triamilide antibiotic, in cattle. Veterinary Therapeutics. 2004; 5: 60–74. 15150731

[41] Rapp RP. Pharmacokinetics and pharmacodynamics of intravenous and oral azithromycin: enhanced tissue activity and minimal drug interactions. The Annals of Pharmacotherapy. 1998; 32: 785–793. 10.1345/aph.17299 9681095

[42] Zhou Q, Zhang G, Wang Q, Liu W, Huang Y, Yu P, et al Pharmacokinetic/Pharmacodynamic modeling of tulathromycin against *Pasteurella multocida* in a porcine tissue cage model. Frontiers in Pharmacology. 2017; 8: 1–11. 10.3389/fphar.2017.00001 28701951 PMC5487385

[43] Benchaoui HA, Nowakowski M, Sherington J, Rowan TG, Sunderland SJ. Pharmacokinetics and lung tissue concentrations of tulathromycin in swine. Journal of Veterinary Pharmacology and Therapeutics. 2004; 27: 203–210. 10.1111/j.1365-2885.2004.00586.x 15305848

[44] Burch DGS. Antimicrobial concentrations in plasma and lung and their relationships to bacterial respiratory infections. The Pig Journal. 2010; 63: 34–49.

[45] Evans NA. Tulathromycin: an overview of a new triamilide antibiotic for livestock respiratory disease. Veterinary Therapeutics. 2005; 6: 83–95. 16094557

[46] Singhvi SM, Heald AF, Schreiber EC. Pharmacokinetics of cephalosporin antibiotics: protein-binding considerations. Chemotherapy 1978; 24: 121–133. 10.1159/000237771 657874

[47] Zeitlinger MA, Sauermann R, Traunmuller F, Georgopoulos A, Muller M, Joukhadar C. Impact of plasma protein binding on antimicrobial activity using time-killing curves. Journal of Antimicrobial Chemotherapy. 2004; 54: 876–880. 10.1093/jac/dkh443 15472003

[48] Zeitlinger MA, Derendorf H, Mouton JW, Cars O, Craig WA, Andes D, et al Protein binding: do we ever learn? Antimicrobial Agents and Chemotherapy. 2011; 55: 3067–3074. 10.1128/AAC.01433-10 21537013 PMC3122431

[49] Dellit TH, Owens RC, McGowan JE, Gerding DN, Weinstein RA, Burke JP, et al Infectious Diseases Society of America and the Society for Healthcare Epidemiology of America guidelines for developing an institutional program to enhance antimicrobial stewardship. Clinical Infectious Diseases. 2007; 44: 159–177. 10.1086/510393 17173212

